# Tissue Morphogenesis Through Dynamic Cell and Matrix Interactions

**DOI:** 10.1146/annurev-cellbio-020223-031019

**Published:** 2023-06-14

**Authors:** Di Wu, Kenneth M. Yamada, Shaohe Wang

**Affiliations:** 1 Janelia Research Campus, Howard Hughes Medical Institute, Ashburn, Virginia, USA; 2 Cell Biology Section, National Institute of Dental and Craniofacial Research, National Institutes of Health, Bethesda, Maryland, USA

**Keywords:** cell adhesion, interfacial tension, extracellular matrix, basement membrane, integrin, cadherin

## Abstract

Multicellular organisms generate tissues of diverse shapes and functions from cells and extracellular matrices. Their adhesion molecules mediate cell-cell and cell-matrix interactions, which not only play crucial roles in maintaining tissue integrity but also serve as key regulators of tissue morphogenesis. Cells constantly probe their environment to make decisions: They integrate chemical and mechanical information from the environment via diffusible ligand- or adhesion-based signaling to decide whether to release specific signaling molecules or enzymes, to divide or differentiate, to move away or stay, or even whether to live or die. These decisions in turn modify their environment, including the chemical nature and mechanical properties of the extracellular matrix. Tissue morphology is the physical manifestation of the remodeling of cells and matrices by their historical biochemical and biophysical landscapes. We review our understanding of matrix and adhesion molecules in tissue morphogenesis, with an emphasis on key physical interactions that drive morphogenesis.

## INTRODUCTION

Multicellular organisms occupy diverse ecological niches by generating functionally diverse tissues. Their tissues vary dramatically in shape, ranging from flat fish scales to spherical eyeballs and branched mammalian lungs. The building blocks of tissues are cells and extracellular matrices (ECMs), which are held together by adhesion molecules mediating cell-cell or cell-matrix associations. Intriguingly, the emergence of many ECM molecules during evolution coincides with multicellularity, highlighting their importance in tissue formation (Özbek et al. 2010).

Tissue morphogenesis is a bottom-up process. It usually begins with small numbers of cells and simple geometry, which become elaborated into complex terminal morphologies. Control of tissue morphogenesis is distributed intrinsically, with each cell being an autonomous controller, making tissues more robust to damage of small numbers of cells. In principle, cells may contribute to tissue morphogenesis by cell proliferation, death, shape change, and rearrangement, with the importance of each differing dramatically in different contexts. In plants, tissue morphogenesis largely arises from patterned cell proliferation since most plant cells do not move. In contrast, animal tissues can change shape substantially without cell proliferation. For example, the bean-shaped *Caenorhabditis elegans* embryo elongates fourfold to become rod shaped, the *Drosophila* ventral nerve cord shortens 2.8-fold during embryogenesis, and ascidian notochord cells morph from a monolayer to a slender rod by convergent extension, all without cell proliferation (Chisholm & Hardin 2005, Jiang & Smith 2007, Karkali et al. 2022). Nevertheless, animal tissue morphogenesis more commonly involves multiple cellular mechanisms.

Pattern formation at the tissue level requires coordination of cell and ECM components. This coordination can be chemical or mechanical in nature. Chemical coordination is usually mediated by diffusible signaling molecules, often termed morphogens in the context of morphogenesis. Morphogen gradients can arise when producer cells are initially nonuniform. More strikingly, Turing’s (1952) reaction-diffusion model explains how morphogen patterns can arise from an initially uniform system, where symmetry breaking can occur by selective amplification of noise in a chemical system at unstable equilibrium. For generating stable patterns in a two-component reaction-diffusion system, one component is an activator while the other is a faster-diffusing inhibitor (Gierer & Meinhardt 1972). In tissues, a source of noise is the stochasticity of gene expression due to low copy numbers of DNA and regulatory molecules (Elowitz et al. 2002). Turing models can successfully explain pattern formation in many biological systems, including zebrafish stripes, mouse palate ridges, mouse limb digits, and tetrapod limb joints (Asai et al. 1999, Economou et al. 2012, Raspopovic et al. 2014, Scoones & Hiscock 2020). Reviews of Turing pattern formation include those by Bailles et al. (2022) and Hiscock & Megason (2015).

Mechanical coordination of morphology shifts tissues toward a mechanically more stable configuration. It varies greatly due to differing forces and mechanical properties of the cells and matrices of each tissue. For example, apical constriction bends an epithelial layer to release tension built up by actomyosin contraction, which is critical for neural tube closure and *Drosophila* gastrulation (Martin 2020, Nikolopoulou et al. 2017). Mechanical instability induced by differential growth of tissue compartments causes buckling or wrinkling to release accumulated growth pressure to drive intestinal villi formation, cerebral cortex folding, and intestinal looping morphogenesis (Savin et al. 2011, Shyer et al. 2013, Tallinen et al. 2016). Local mechanical restraint by smooth muscle or collagen matrix accumulation helps guide bifurcation of mouse lungs and mammary glands (Kim et al. 2015, Nerger et al. 2021). Hydrostatic pressure from osmotic swelling of locally synthesized hyaluronan drives invagination of the otic epithelium to initiate zebrafish semicircular canal formation (Munjal et al. 2021). Characteristic adhesion preferences of distinct cell types toward other cells or the basement membrane matrix drive robust patterning of the developing zebrafish spinal cord and budding morphogenesis of mouse embryonic salivary glands (Tsai et al. 2020, Wang et al. 2021a).

The mechanical interactions shaping tissue morphology are guided by biochemical signaling, which can regulate the number, shape, or position of cells, as well as the density, components, or distribution of ECMs. Adhesion molecules, which physically connect all tissue components together, are uniquely positioned to serve as regulators of tissue morphogenesis. Below, we review common themes of adhesion in morphogenesis, how different families of adhesion molecules mediate adhesion, and the biophysical mechanisms underlying morphogenesis.

## ADHESION IN MORPHOGENESIS

**Figure 1:**
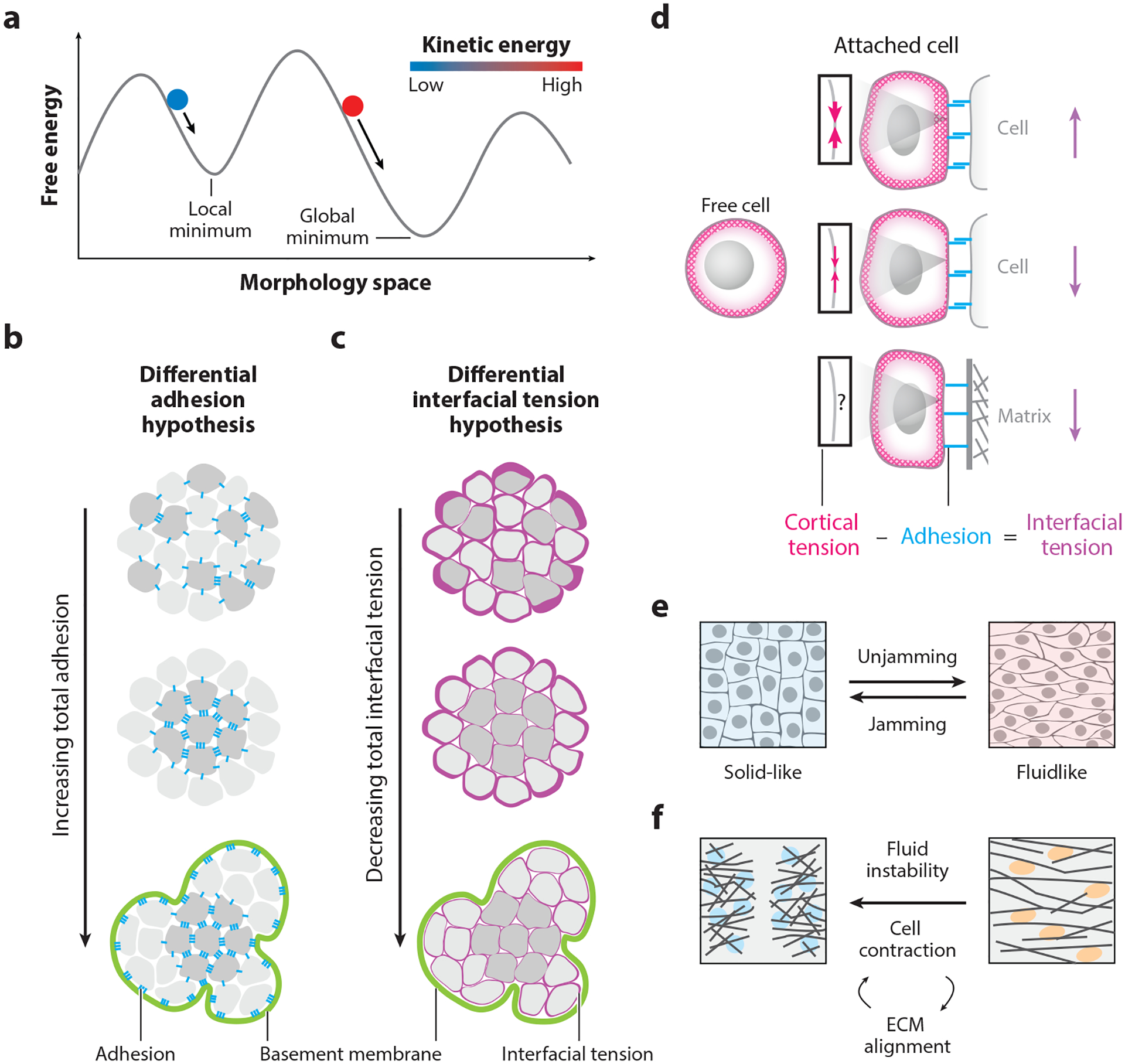
Biophysical principles of tissue morphogenesis. (*a*) Each tissue explores morphology space. Directional change is determined by free energy minimization, while the speed of change is controlled by the kinetic energy. (*b*,*c*) Two theories explaining cell sorting during tissue morphogenesis. (*d*) Cortical tension changes when a cell attaches to another cell or matrix. (*e*) Jamming and unjamming transitions during tissue morphogenesis decrease or increase the kinetic energy, respectively. (*f*) Tissue patterning by fluid instability can be generated from reciprocal cell contraction and extracellular matrix (ECM) alignment.

Tissue morphogenesis requires morphological changes over time. At any point, tissue morphology is determined by the position, size, and shape of each cell and ECM element, which may be represented by a list of numbers, or a vector in high-dimensional space. The ensemble of all possible tissue morphologies occupies a subset of this high-dimensional space, where neighboring vectors represent morphologies with minute changes. Each morphology vector has a free energy that measures its relative stability and a kinetic energy that determines its relative pliability. Both energy values of a tissue are partly determined by cell adhesion. A general principle of tissue morphogenesis is to minimize systemic free energy with constraints from the initial state and tissue pliability (Figure 1*a*). Conceptually, tissue morphology at a later point depends on the direction and rate of changes at an earlier state. The direction of change is biased by the local free energy landscape, preferentially proceeding down the steepest hill. Importantly, however, unlike a regular thermodynamic system, tissues can contribute metabolic energy to help them go uphill. The rate of change is set by the kinetic energy correlating with the mobility and mechanical properties of cells and ECM components. Generally, tissue morphology changes faster when cells move faster and ECM components are softer.

The classical differential adhesion hypothesis (DAH) can successfully explain cell sorting patterns of tissues with high kinetic energy (Steinberg 1963). The DAH proposes that cell rearrangement within a tissue aims to minimize the total surface free energy, which decreases as overall cell-cell adhesion strength increases (Figure 1*b*). The most profound prediction of the DAH is that robust sorting of different cell types can arise from quantitative differences between their adhesion strengths, whereas other models assume qualitative selectivity of adhesion (e.g., Moscona 1957). The DAH can successfully explain many cell sorting behaviors, including enveloping of more-adhesive cells by less-adhesive cells. Mutual envelopment of five chick embryonic tissues precisely follows the order of their tissue surface tensions used as a measure of cell adhesion strength (Foty et al. 1996). Similarly, the surface tension of *Hydra* endoderm is higher than the external ectoderm, and cells expressing more cadherins (a major cell-cell adhesion molecule; see the section titled Cell-Cell Adhesion) are enveloped by cells expressing fewer (Cochet-Escartin et al. 2017, Steinberg & Takeichi 1994). Intriguingly, tissue surface tension correlates linearly with experimental expression levels of cadherin molecules (Foty & Steinberg 2005).

Debates around whether adhesion is the dominant contributor to tissue surface tension catalyzed the development of the differential interfacial tension hypothesis (DITH) (Brodland 2002, Harris 1976, Tsai et al. 2022). The DITH includes the DAH concept of minimizing surface free energy, but it adds contributions of cell cortical tension to cell adhesion (Figure 1*c*,*d*). Measured by parallel plate compression or micropipette aspiration assays, tissue surface tensions of cell aggregates or embryonic tissues from chick, zebrafish, or *Hydra* are all ~10^−3^ N/m (Cochet-Escartin et al. 2017, Foty & Steinberg 2005, Foty et al. 1996, Schötz et al. 2008). In contrast, the average binding energy of cadherins is ~10^−19^ Joules, corresponding to an adhesion-based tension of ~10^−7^ N/m at a density of 10 cadherins/μm^2^ (Katsamba et al. 2009, Sivasankar et al. 1999). This four-orders-of-magnitude discrepancy strongly argues against direct cadherin binding as the dominant contributor to tissue surface tension, and the cell sorting behavior of zebrafish germ layer progenitor cells correlates better with cell cortical tension than adhesion properties (Krieg et al. 2008). Thus, cell cortical tension is proposed to be the dominant contributor to tissue surface tension (Amack & Manning 2012).

If cadherin binding only accounts for approximately one ten-thousandth of tissue surface tension, how can we explain the linear correlation between tissue surface tension and cadherin expression levels (Foty & Steinberg 2005)? One idea is that cadherin’s main role is transmembrane signaling to reduce local cortical tension (Amack & Manning 2012, Maruthamuthu et al. 2011). However, this would require cadherin to be the only missing molecule of a signaling cascade that scales linearly with cadherin abundance over a large dynamic range (Foty & Steinberg 2005). Alternatively, cadherin binding may enable binding of additional nearby surface molecules to contribute more adhesion energy. Interestingly, adhesion and cortical tension could contribute similarly to surface tension–generated cell power, measured as the rate of work done against gravity when a toroid-shaped cell aggregate climbs up a conical peg while shrinking the tissue surface (Youssef et al. 2011). First, the power output of a fibroblast toroid is reduced by ~50% when treated with Rho kinase inhibitor, which inhibits myosin contractility. Second, when the work against gravity calculated in this study is corrected for buoyancy, the cadherin density (~12 cadherins/μm^2^) appears realistic.

The DITH provides a broader and more generalizable framework than the DAH. Unlike the DAH, which assumes uniform adhesiveness and stiffness around the cell surface, the DITH embraces intracellular asymmetry. The interfacial tension at different surface locations of the same cell can vary significantly depending on the local balance between adhesion and cortical tension. Adhesion seeks to expand the interface, while cortical tension seeks to shrink the interface. Hence, interfacial tension decreases with stronger adhesion and weaker cortical tension. Varying levels of cortical tension at the cell-medium interface in zebrafish germ layer progenitor cells are a critical determinant of their sorting behavior (Krieg et al. 2008). While some adhesive signals to the cell reduce both cortical tension and interfacial tension (e.g., classical cadherins), other adhesion signaling can increase cortical tension and interfacial tension, for example, clustered protocadherins and Eph/ephrin binding (Tsai et al. 2022). Different cell types can acquire homotypic or heterotypic adhesive preference through combinatorial expression of different adhesion molecules to ensure robust pattern formation, such as the stereotypical stripe-like pattern of neural progenitors in zebrafish embryos (Tsai et al. 2020).

While free energy changes guide the direction of tissue morphogenesis, the rate of change depends mainly on cell dynamics and tissue rheology, that is, how materials react to forces (Petridou & Heisenberg 2019). Tissues can be quite heterogeneous with regions that are qualitatively liquid like or solid like. Compared to solid-like tissues, liquid-like tissues often have lower cell density, more elongated cell shapes, and higher cell motility. Recently, the terms jamming and unjamming have become popular to describe transitions between liquid-like and solid-like states of cellular tissues, which often involve cell adhesion changes (Blauth et al. 2021, Petridou & Heisenberg 2019, Tambe & Fredberg 2015) (Figure 1*e*). Jamming transition can kinetically trap 3D mixed cultures of different cell lines in a solid-like state, preventing them from reaching the predicted free energy minimum (Pawlizak et al. 2015). During zebrafish body axis elongation, jamming transition of the liquid-like posterior tissue at the extending end to a solid-like state is critical for mechanically supporting unidirectional axis extension (Mongera et al. 2018). Conversely, unjamming transition facilitates cell rearrangement and blastoderm spreading in zebrafish embryogenesis (Petridou et al. 2019). Epithelial-to-mesenchymal transition (EMT) is a classical type of unjamming transition that underlies neural crest cell migration and cancer metastasis (Ilina et al. 2020, Kerosuo & Bronner-Fraser 2012). Interestingly, however, unjamming transition and partial EMT cause distinct changes in airway epithelial cells, indicating that unjamming transition may arise from EMT-independent pathways (Mitchel et al. 2020). Moreover, jamming and unjamming of adjacent tissues can be mechanically coupled. For example, in *Xenopus* embryos, jamming and stiffening of the underlying mesoderm trigger EMT and collective migration of neural crest cells (Barriga et al. 2018). In summary, tissue rheology changes, such as jamming and unjamming transitions, can play both permissive and regulatory roles in tissue morphogenesis.

Both interfacial free energy and tissue rheology patterns can be strongly influenced by ECM composition and cell-ECM interactions (Chaudhuri et al. 2020). For example, in vivo epithelial tissues are often demarcated by the basement membrane, a thin dense layer of ECM (see the section titled Basement Membrane). Unlike a cell-medium interface with high interfacial tension that tissues strive to minimize, cell-matrix interfaces at basement membranes could be the preferred type of interface with lower interfacial tension for tissues to expand (Figure 1*b*,*c*). Recent work reveals that preferential cell-matrix adhesion between surface epithelial cells and basement membrane ECMs is indeed a key driver of budding morphogenesis in mouse embryonic salivary glands (Wang et al. 2021a). During the branching morphogenesis of chick embryonic lungs, unjamming of nearby mesenchymal cells may help move locally accumulated ECMs to facilitate epithelial branch extension (Spurlin et al. 2019). Unjamming or fluidization sometimes requires reciprocal cell-ECM rearrangements. For example, patterning of regularly spaced feather follicles in chick embryos arises from supracellular fluidity of aligned mesenchymal cells and ECM fibers in a cell-ECM composite layer (Palmquist et al. 2022) (Figure 1*f*).

## CELL-CELL ADHESION

While plant cells attach to each other through cell walls, animal cells can directly adhere to each other via transmembrane proteins, including cadherins, protocadherins, immunoglobulin cell-adhesion molecules, occludins, and claudins. The extracellular domains of these proteins mediate homophilic or heterophilic binding between cells, whereas their intracellular domains often link to the cytoskeleton through adaptor proteins. Below, we focus on the best-studied classical cadherins playing critical roles in tissue morphogenesis. Reviews of other molecules include those by Cummins (2012), Kowalczyk & Green (2013), Tsai et al. (2022), and Tsukita et al. (2019).

**Figure 2:**
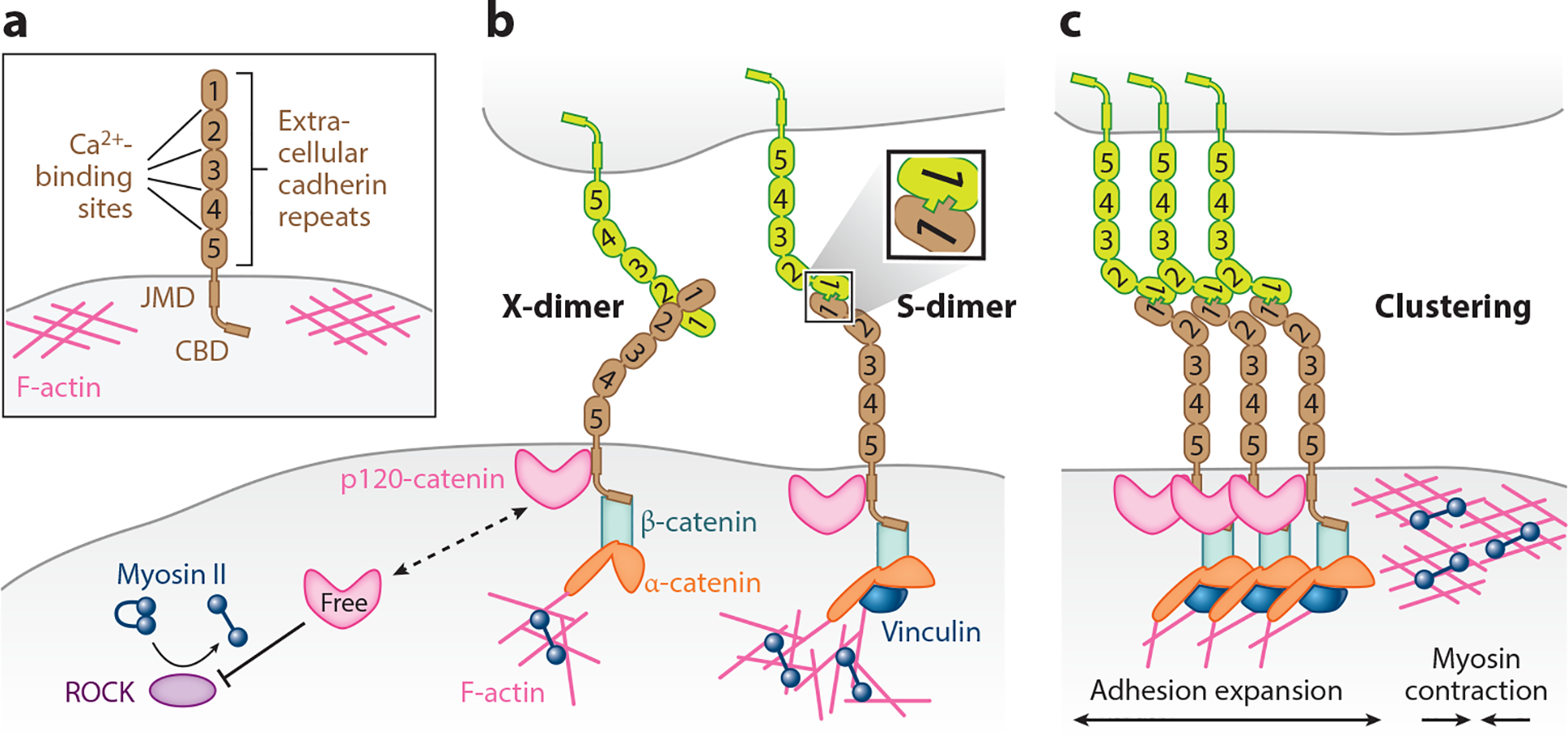
Cell-cell adhesion mediated by classical cadherins. (*a*) Domain structure of classical cadherins. (*b*) Extracellular and intracellular interactions of cadherins. X-dimer and S-dimer are two conformations of *trans* dimers. (*c*) Cadherin dimers can cluster via *cis* interactions. Expansion of the adhesion surface is driven by adjacent actomyosin contraction. Abbreviations: CBD, catenin-binding domain; JMD, juxtamembrane domain.

Classical cadherins are calcium-dependent homophilic binding molecules that are conserved throughout metazoans (Hulpiau & van Roy 2009). Their extracellular domains consist of five extracellular cadherin (EC) repeats, and three calcium ions bind to each EC-EC interface to stabilize the structure (Boggon et al. 2002) (Figure 2*a*). Cadherins on neighboring cells can bind in *trans* to mediate adhesion, whereas cadherins on the same cell can bind in *cis* to facilitate clustering (Boggon et al. 2002, Harrison et al. 2011) (Figure 2*b*,*c*). The *trans* dimer can adopt an intermediate X-dimer conformation that transitions to a more stable S-dimer (strand-swap dimer) conformation (Harrison et al. 2010). While the X-dimer is mediated by residues near the EC1-EC2 interface, the S-dimer is mediated by strand-swapping between EC1 domains, where one (type I cadherins) or two (type II cadherins) conserved tryptophan residues insert into the hydrophobic pocket of the interacting partner (Hulpiau & van Roy 2009). Interestingly, the X-dimer exhibits catch-bond behavior, where the bond lifetime increases with greater pulling force until a threshold of ~20 piconewtons (pN) is reached (Rakshit et al. 2012). The catch-bond property of X-dimers is likely important for stabilizing initial cell-cell contacts.

The cytoplasmic tail of classical cadherins harbors two distinct binding sites for two catenins, p120- and β-catenin, which have distinct functions (Figure 2*a*,*b*). For E-cadherin, the best-studied classical cadherin, binding of p120-catenin to the juxtamembrane domain of the cytoplasmic tail promotes its stability by masking residues mediating Hakai-dependent ubiquitination and clathrin-mediated endocytosis (Fujita et al. 2002, Ishiyama et al. 2010). Free cytoplasmic p120-catenin, but not cadherin-bound p120-catenin, can inhibit RhoA activity that promotes actin polymerization and myosin contractility (Anastasiadis et al. 2000) (Figure 2*b*). In addition, p120-catenin can interact with PLEKHA7, which in turn binds to the microtubule minus-end binding protein, CAMSAP3, linking adhesion to the microtubule cytoskeleton (Meng et al. 2008). The catenin-binding domain of the cytoplasmic tail interacts with β-catenin, which in turn binds α-catenin, providing a bridge to the actin cytoskeleton (Takeichi 2014). Interestingly, the binding of α-catenin within the minimal cadherin-catenin complex to actin filaments also exhibits catch-bond behavior, with a peak lifetime at ~10 pN (Buckley et al. 2014). Moreover, α-catenin under tension exposes a binding site for vinculin, which cooperates with α-catenin to reinforce interactions with actin filaments (Xu et al. 2020) (Figure 2*b*). Recent work reveals a critical role for asymmetric accumulation of vinculin in mitotic versus neighboring cells for maintaining epithelial integrity (Monster et al. 2021).

Cell-cell adhesion can regulate tissue morphogenesis via interfacial tension or cell motility. While adhesive binding itself reduces interfacial tension, varying cortical tension may produce a net increase or decrease of interfacial tension. For example, binding of clustered protocadherins increases cortical tension and net interfacial tension by inhibiting Pyk2 and focal adhesion kinase (FAK) for neurite self-avoidance from the same neuron (Tsai et al. 2022). In contrast, binding of classical cadherins decreases cortical tension and net interfacial tension for cell sorting in morphogenesis (Maître et al. 2012, Yamada & Nelson 2007). In zebrafish spinal cord morphogenesis, combinatorial expression of various cadherins in different cell types ensures robust formation of a stripe-like pattern (Tsai et al. 2020). Furthermore, adhesion coding has been leveraged to facilitate precise patterning of synthetic mouse embryos or to program multicellular assembly (Bao et al. 2022, Stevens et al. 2023). In general, cell motility inversely correlates with cadherin expression. A hallmark of EMT is the repression of cadherin expression by EMT transcription factors since cadherin-mediated cell-cell adhesion inhibits cell motility (Kerosuo & Bronner-Fraser 2012). Conversely, the jamming transition of posterior tissue during zebrafish body axis elongation requires N-cadherin-mediated adhesion (Mongera et al. 2018).

## CELL-MATRIX ADHESION

### Extracellular Matrix Overview

**Figure 3:**
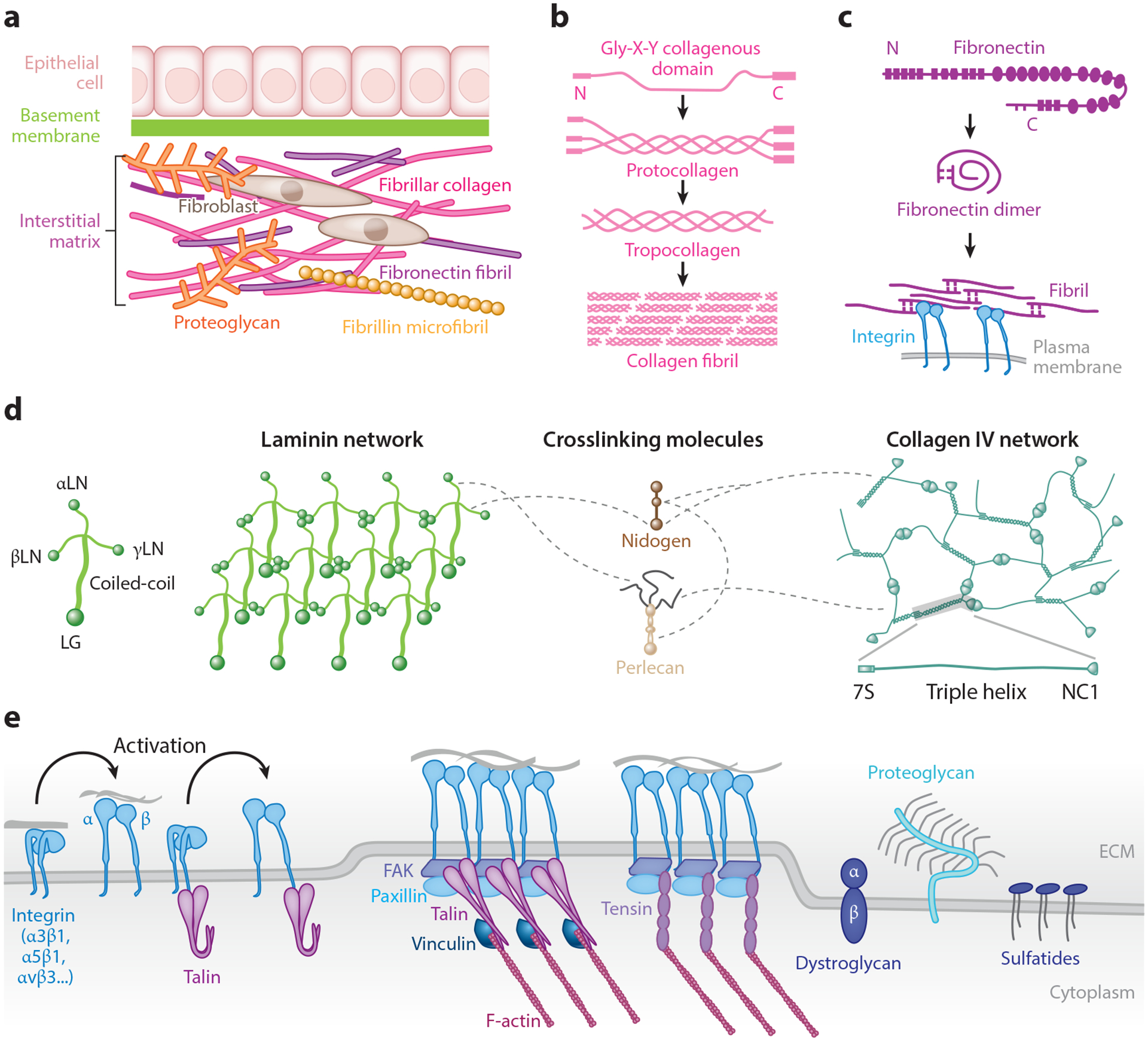
Extracellular matrices and their receptors. (*a*) Basement membrane and interstitial matrix are two major types of extracellular matrix. (*b*,*c*) Assembly of the collagen fibril (*b*) or fibronectin fibril (*c*). (*d*) Components of the basement membrane. Dashed lines indicate some known interactions. (*e*) Matrix receptors on the cell surface and adaptor/signaling proteins binding to the cytoplasmic tail of integrins. Abbreviations: 7S, N-terminal domain with a sedimentation coefficient of 7S as a tetramer; C, C terminus; ECM, extracellular matrix; FAK, focal adhesion kinase; LG, laminin globular domain; LN, laminin N-terminal domain; N, N terminus; NC1, noncollagenous 1.

The ECM in plants and animals contains two major classes of macromolecules: polysaccharides and proteins. Plant ECM consists mainly of cell walls composed of polysaccharides, including cellulose, hemicellulose, and pectins (Keegstra 2010). Animal ECMs are much more diverse in form but largely fall into two categories: interstitial matrix and basement membrane (Cruz Walma & Yamada 2020, Frantz et al. 2010) (Figure 3*a*). This review does not cover mineralized ECMs, including bones, teeth, and specialized ECMs such as chitin-rich insect exoskeletons.

### Interstitial Matrix

Glycosaminoglycans (GAGs) are polymeric glycans found in the ECM of most metazoans (Yamada et al. 2011). GAG chains have type-specific disaccharide repeats. Vertebrates have four major types of GAGs: heparin/heparan sulfate, chondroitin/dermatan sulfate, keratan sulfate, and hyaluronan (which lacks sulfates). All GAGs are highly negatively charged. Their negative charges enable GAGs to bind positively charged amino acids in proteins via ionic interactions, which dominate GAG-protein interactions. Consequently, the sulfation patterns of GAGs, known as the sulfation code, can significantly affect their substrate-binding specificity or affinity (Soares da Costa et al. 2017). Hyaluronan can generate hydrostatic pressure to drive invagination of the otic epithelium during zebrafish semicircular canal morphogenesis (Munjal et al. 2021).

Sulfated GAGs attach to the protein core of proteoglycans, whereas hyaluronan lacks either a protein core or sulfation (Dicker et al. 2014). The protein cores of proteoglycan define three major families: the small leucine-rich proteoglycans (SLRPs), modular proteoglycans, and cell-surface proteoglycans (Schaefer & Schaefer 2009). SLRP protein cores have leucine-rich repeats that commonly mediate protein-protein interactions. Examples of SLRPs include decorin and biglycan, containing chondroitin sulfate/dermatan sulfate chains that can bind transforming growth factor β (TGFβ) to regulate development, for example, of skeletal muscle (Brandan et al. 2008). Biglycan plays important roles in bone formation by modulating bone morphogenetic protein-4 (BMP-4)-induced osteoblast differentiation (Chen et al. 2004). Aggrecan is a hyaluronan-binding modular proteoglycan that is mainly expressed in brain and cartilage. Aggrecan mutations cause cartilage formation defects in both mouse and human (Gleghorn et al. 2005, Watanabe et al. 1994).

Proteins within the ECM often form oligomers that further assemble into fibrils or networks. Alternatively, they may serve as cross-linkers by interacting with multiple other ECM molecules. Fibrillar collagens are found in all animals, including sponges (Özbek et al. 2010). They commonly have a triple helix-forming collagenous domain flanked by two noncollagenous domains. The collagenous domain of mammalian fibrillar collagens is ~1,000 amino acids long and is composed of uninterrupted repeats of the Gly-X-Y motif that is critical for triple helix formation (Kadler et al. 2007) (Figure 3*b*). After triple helix formation, the noncollagenous domains at the N and C termini are removed by propeptidases to enable higher-order assembly into fibrils up to 500 nm in diameter and 1 cm in length (Shoulders & Raines 2009). Collagen fibrils can be covalently cross-linked by lysyl oxidase to enhance fibril strength and stability, as well as tissue stiffness (Lucero & Kagan 2006). The mechanical strength of collagen fibers constrains the bifurcation angle of growing epithelial tips during branching morphogenesis of the mouse mammary gland (Nerger et al. 2021). Interactions between collagen fibers and contractile mesenchymal cells can generate supracellular fluidity underlying morphogenetic patterning of chick feather follicles (Palmquist et al. 2022).

Fibrillin microfibrils are also conserved throughout metazoans (Özbek et al. 2010). Fibrillin can bind fibulin, latent TGFβ-binding proteins (LTBPs), BMP, lysyl oxidase, tropoelastin, perlecan, fibronectin, and so on. (Thomson et al. 2019). Multimodal binding by fibrillin may underlie a scaffolding role for microfibrils in guiding tropoelastin alignment and cross-linking during elastic fiber assembly (Muiznieks et al. 2010). In fact, double knockout of fibrillin-1 and −2 genes in mice causes mid-gestation death with a poorly developed aortic media, which is normally rich in elastic fibers (Carta et al. 2006).

Elastic fibers are the dominant contributor of ECM elasticity (Muiznieks et al. 2010). They have minimal turnover and are extremely durable, as exemplified by elastic fibers in the arterial wall that can withstand over two billion cycles of stretch and relaxation (Davis 1993, Rauscher & Pomès 2017). Ninety percent of the elastic fiber is elastin, a polymer made from tropoelastin. The excellent elasticity of elastin fibers arises from a combination of hydrophobic effects and conformational chain entropy of cross-linked disordered chains (Fawzi 2020, Rauscher & Pomès 2017). Elastin-null mice die shortly after birth from obstructive arterial disease, along with distal lung morphogenesis defects (Li et al. 1998, Wendel et al. 2000).

Fibronectin can generate another important ECM fiber bound by multiple integrins (see the section titled Matrix Receptors) and ECM molecules, including collagen, fibrin, heparan sulfate, and other fibronectin molecules (Pankov & Yamada 2002). Fibronectin matrix assembly is a tightly regulated cell-mediated process requiring actomyosin contractility and integrin dynamics (Huet-Calderwood et al. 2022, Pankov & Yamada 2002) (Figure 3*c*). Fibronectin matrix assembly can be mediated by inward translocation of α5β1 integrin–containing fibrillar adhesions from stable peripheral focal adhesions containing αVβ3 integrins (Geiger & Yamada 2011, Pankov et al. 2000). However, cells on basement membrane components instead assemble matrix by inward-sliding focal adhesions driven by an actomyosin contractile winch (Lu et al. 2020). This contractile-winch model can explain the enrichment of fibronectin matrix at basement membranes that is critical for multiple morphogenetic events in vertebrate embryos, including amphibian mesodermal cell migration during gastrulation (Boucaut et al. 1984). Later in embryogenesis, fibronectin plays critical roles in branching morphogenesis of multiple mammalian organs, including salivary gland, lung, and kidney (Sakai et al. 2003).

How do these different ECM components contribute to the mechanical properties of cellular microenvironments? A simplified view is that GAGs and proteins in the ECM are responsible for its compressive and tensile strength, respectively. GAGs form hydrogels that are important for resisting compressive forces by swelling in water due to their hydrophilicity, electrostatic repulsion of negative charges, and osmotic pressure of counterions (Scott 2003). GAG hydrogels likely confer poroelasticity to some ECMs (Chaudhuri et al. 2020). While GAGs help ECMs resist compressive forces, protein fibrils and networks are responsible for withstanding tensile forces. In addition to noncovalent interactions, ECM fibrils and networks have covalent crosslinks generated by lysyl oxidase and transglutaminase (Lorand & Graham 2003, Lucero & Kagan 2006). Increased expression and aberrant crosslinking of fibronectin and collagen fibrils are generally responsible for the increased stiffness and reduced elasticity of aged, fibrotic, and tumor tissues (Frantz et al. 2010).

### Basement Membrane

The basement membrane is a dense thin layer of ECM marking the boundary of many tissues, including epithelia, muscle, and adipose tissues (Jayadev & Sherwood 2017). It consists of a laminin network, a collagen IV network, and crosslinking molecules, including nidogen and perlecan (Figure 3*d*). In addition, proteomic analyses identify fibronectin, tenascin C, TGFBI (TGFβ induced), fibrillin, agrin, and collagens XV and XVIII in basement membranes from multiple tissues (Randles et al. 2017). Basement membranes are present in all bilaterian and some nonbilaterian animals (Fidler et al. 2017).

Laminins are heterotrimers with α, β, and γ subunits. Laminin heterotrimers are commonly cross shaped with one long coiled-coil arm and three short arms, or Y shaped if missing the α short arm. Their coiled-coil domains mediate trimer assembly, while the laminin N-terminal (LN) and laminin globular (LG) domains are mainly responsible for network formation and cell adhesion, respectively (Yurchenco 2011). The C-terminal LG domains of laminin α interact with multiple cell surface receptors, including integrins, dystroglycans, and sulfatides (Yurchenco 2011). Besides playing a primary role in laminin polymerization, LN domains also provide additional cell-adhesion sites for integrins and sulfatides. Laminin network formation is the critical initial step for basement membrane assembly (Smyth et al. 1999). Genetic ablation of either laminin β1 or laminin γ1 in mice results in the absence of basement membranes and early embryonic death, with absent or disorganized collagen IV, nidogen, and perlecan (Miner et al. 2004, Smyth et al. 1999).

Besides the laminin network, collagen IV forms another covalently cross-linked network to add tensile strength to the basement membrane. Type IV collagens are heterotrimers that are ~480 kDa in size. Unlike fibrillar collagens, the noncollagenous domains of collagen IV are not excised but instead mediate network formation (Cummings et al. 2016, Khoshnoodi et al. 2008, Vanacore et al. 2009). The collagenous domains have both terminal and extensive lateral associations (Yurchenco & Ruben 1987). Unlike laminins, knockout of collagen IV does not prevent basement membrane formation and function in early embryos, but it causes basement membrane integrity defects and postimplantation lethality due to Reichert’s membrane rupture (Pöschl et al. 2004). Interestingly, *Drosophila* egg chambers elongate in a direction perpendicular to circumferentially aligned collagen IV fibrils in the surrounding basement membrane (Haigo & Bilder 2011). Genetic collagen IV knockout in *Drosophila* results in a rounder egg chamber consistent with loss of tensile strength by weakening this molecular corset (Haigo & Bilder 2011, Ramos-Lewis & Page-McCaw 2019).

The laminin and collagen IV networks of basement membranes are cross-linked by multiple ECM molecules, including nidogen and perlecan. Nidogen, also termed enactin, is a glycoprotein that binds to both laminin and collagen IV (Yurchenco 2011). Mammals have two nidogens that are 130–150 kDa in size. Genetic knockout of both nidogens in mice causes perinatal lethality from tissue-specific basement membrane defects without affecting early embryogenesis (Bader et al. 2005). In fact, kidneys can develop normally without nidogens, whereas lung development is only delayed at a late stage (Bader et al. 2005). Genetic ablation of the single *Drosophila* nidogen gene does not affect overall organogenesis or viability, and nidogen deletion in the nematode *C. elegans* does not cause obvious phenotypes (Dai et al. 2018, Kang & Kramer 2000).

Perlecan is a heparan sulfate proteoglycan found in nearly all basement membranes and in interstitial matrices, including cartilage (Farach-Carson & Carson 2007, Pozzi et al. 2017). Perlecan knockout mice die from severe cartilage defects or basement membrane disruption at sites of elevated mechanical stress (Arikawa-Hirasawa et al. 1999, Costell et al. 1999). These mouse knockout phenotypes suggest that perlecan and collagen IV help to maintain basement membrane mechanical strength, yet genetic studies in *Drosophila* suggest that they can have opposing roles (Pastor-Pareja & Xu 2011, Ramos-Lewis & Page-McCaw 2019). Loss of collagen IV and perlecan overexpression both result in rounder egg chambers and extended ventral nerve cords, indicative of decreased stiffness, whereas increased collagen IV levels and loss of perlecan cause phenotypes consistent with increased basement membrane stiffness (Ramos-Lewis & Page-McCaw 2019). Whether these apparently counteracting roles of perlecan and collagen IV are purely mechanical or involve signaling is not yet clear.

Basement membranes can guide tissue morphogenesis in different ways. The *Drosophila* egg chamber molecular corset involves anisotropic stiffness of the basement membrane (Ku et al. 2023). Atomic force microscopy reveals that the central constricting region becomes stiffer than anterior and posterior regions as elongation begins (Crest et al. 2017). A mechanical role of the basement membrane has also been identified in morphogenesis of stratified epithelial tumors, where softening and stiffening of the basement membrane promote the generation of different tumor morphologies (Fiore et al. 2020). Additionally, the basement membrane and its tight association with cells can change the free energy landscape by reducing interfacial tension at the cell–basement membrane contact surface (Figure 1*c*). This mechanism plays critical roles in driving the budding morphogenesis of developing mouse salivary glands (Wang et al. 2021a).

### Extracellular Matrix Dynamics

The importance of ECM dynamics for animal development is engraved in evolution. Vertebrates have three major families of ECM-remodeling enzymes, including the matrix metalloproteinases (MMPs), a disintegrin and metalloproteases (ADAMs), and a disintegrin and metalloproteinase with thrombospondin motifs (ADAMTSs); MMPs and ADAMTSs are conserved throughout metazoans (Özbek et al. 2010), and these proteases have been extensively reviewed elsewhere (Kelwick et al. 2015, Primakoff & Myles 2000, Sternlicht & Werb 2001). Overall, they have distinct but often overlapping substrate specificities that together can cleave virtually all ECM proteins. Some ECM proteases are secreted, while others are membrane tethered. Their activities are regulated by transcriptional expression as well as by tissue inhibitors of metalloproteinases (TIMPs) (Nagase et al. 2006). Despite substantial overlapping substrates between different MMPs, knockout of MMP14 alone causes delayed branching morphogenesis of salivary glands in mice (Oblander et al. 2005).

The importance of basement membrane dynamics for morphogenesis is further highlighted by enhanced remodeling at sites undergoing active expansion. For example, numerous basement membrane microperforations appear at the growing tips of mouse embryonic organs, including lungs, kidneys, and salivary glands, to permit tissue expansion (Harunaga et al. 2014). These basement membrane perforations and branching morphogenesis of salivary glands are both inhibited by inhibitors of MMPs and/or ADAMs (Harunaga et al. 2014, Wang et al. 2021a). Similar basement membrane perforations are found at the posterior side of gastrulating mouse embryos to permit primitive-streak extension (Kyprianou et al. 2020). How cells fine-tune the turnover rate and mechanical strength of expanding basement membranes during tissue morphogenesis needs elucidation.

ECM dynamics are not limited to protease-mediated turnover. A recent study in *C. elegans* reveals extensive ECM movements within the basement membrane by fluorescence recovery after photobleaching experiments using many endogenously mNeonGreen-tagged matrix proteins (Keeley et al. 2020). Determining whether similar dynamics are observed in other species and evaluating the functional importance of this dynamic lateral diffusion within the basement membrane remain intriguing problems.

### Matrix Receptors

Cells bind to the ECM via many matrix receptors, including integrins, cell-surface proteoglycans, discoidin domain receptors, dystroglycan, and sulfatides (Figure 3*e*). Integrins and cell-surface proteoglycans are conserved throughout metazoans (Özbek et al. 2010). Many ECM receptors function by linking the ECM to the actin cytoskeleton. Below, we focus on integrins, the best-known ECM receptors with widespread roles in morphogenesis. Reviews of other receptors include those by Afratis et al. (2017), Honke (2013), Leitinger (2014), and Moore & Winder (2010).

Integrins are heterodimers with α and β subunits. Vertebrates have 24 known integrins combined from 18 α and 8 β subunits (Luo et al. 2007). All integrins have a large head containing ligand-binding sites with two transmembrane legs that can link to the cytoskeleton (Figure 3*e*). By ligand specificity, integrins can be broadly classified into arginine-glycine-aspartate (RGD)-binding (all αV integrins, α5β1, α8β1, and αIIbβ3), leucine-aspartate-valine (LDV)-binding (all four β2 integrins, α4β1, α9β1, α4β7, and αEβ7), collagen-/laminin-binding (α1β1, α2β1, α10β1, and α11β1), and selective laminin-binding (α1β1, α2β1, α10β1, and α11β1, plus α3β1, α6β1, α7β1, and α6β4) integrins (Campbell & Humphries 2011).

The ligand-binding affinity of integrins is conformation dependent and regulated from both inside and outside the cell (Hynes 2002, Kechagia et al. 2019). Newly synthesized integrins dimerize in the endoplasmic reticulum in a bent-closed conformation with low ligand-binding affinity. Upon activation, the extracellular domain of integrins acquires an extended-open conformation with high ligand-binding affinity (Figure 3*e*). Talin or kindlin binding to the β integrin cytoplasmic tail induces its separation from the α integrin cytoplasmic tail and subsequent global conformational activation during inside-out signaling. Alternatively, low-affinity ECM binding with low piconewton forces can pull integrins open to activate them outside-in. Activated integrins then tightly bind to the ECM extracellularly and recruit adaptor proteins linking them to the cytoskeleton at their cytoplasmic tails.

Activated integrins often cluster to form focal adhesions or fibrillar adhesions (Doyle et al. 2022). Integrin clustering is energetically favorable by reducing the mechanical load on each integrin, and it restricts integrin lateral diffusion while locally bending the plasma membrane toward ligands to overcome glycocalyx steric barriers (Kechagia et al. 2019). Compared to small, short-lived nascent adhesions, focal adhesions and fibrillar adhesions have extended lifetimes ranging from minutes to hours (Doyle et al. 2022). Fibrillar adhesions are elongated, and tensin, rather than talin, binds to the β integrin cytoplasmic tail for activation (Kechagia et al. 2019).

Clustered integrins recruit and activate many intracellular signaling molecules, including FAK, paxillin, SRC kinase, and extracellular signal-regulated kinase (ERK), to mediate outside-in signaling (Kechagia et al. 2019). Interestingly, recent work has identified a role of molecular phase separation for recruiting focal adhesion components (Wang et al. 2021b). Outside-in signaling causes important downstream effects, including enhanced actin polymerization and altered gene expression by modulating mechanosensitive transcription regulators such as YAP/TAZ or by directly transmitting force from the ECM to the nucleus (Kechagia et al. 2019).

The mechanosensitivity of integrins enables cells to sense their mechanical microenvironment. As in E-cadherin binding, integrin-ligand binding exhibits catch-bond behavior, where the bond lifetime peaks at 20–30 pN (Elosegui-Artola et al. 2016, Kong et al. 2009). This property permits small pulling forces to activate and stabilize integrins, while large pulling forces can destabilize integrins to promote adhesion dynamics. Mechanotransduction mediated by integrins depends on both molecular tension and the type of ligand. Studies using DNA-based tension gauges have established that RGD-binding integrins require a peak tension of ~40 pN to activate cell spreading of multiple cell lines (Wang & Ha 2013). In contrast, fibroblast spreading mediated by α4β1 integrins requires less than 12 pN (Jo et al. 2022). Moreover, fibroblasts polarize and migrate on RGD substrates but not on α4β1-specific leucine-aspartate-valine-proline (LDVP) substrates (Jo et al. 2022). The different tension thresholds depend on tighter binding to paxillin by the cytoplasmic tail of α4 integrin than other integrins, which may trigger integrin-specific actin cytoskeletal remodeling underlying different cellular phenotypes (Liu et al. 1999). Molecular tension sensors could be used to study mechanosensing by other integrins, including laminin-binding integrins.

Integrins are required for many forms of tissue morphogenesis. Despite the apparent functional redundancy of integrins, knockout of individual integrins in mice results in morphogenesis defects ranging from failed gastrulation (β1 subunit) to neural crest apoptosis (α5), skin blistering (α6, β4), and reduced branching morphogenesis of mammary gland or lung (α2, α3) (Hynes 2002). Blocking antibody and genetic studies also demonstrate requirements for integrins in branching morphogenesis of salivary glands and pancreas (Sakai et al. 2003, Sekiguchi et al. 2023, Shih et al. 2016).

Integrins can contribute to tissue morphogenesis via at least three mechanisms. First, integrins are critical for the assembly and reorganization of some ECMs. For example, fibronectin matrix assembly is integrin dependent (Pankov & Yamada 2002). Laminin network assembly, the initial step of basement membrane formation, also often depends on integrins, though sulfatides and dystroglycan can be more important in certain tissues (Yurchenco 2011). Integrins might mediate the reciprocal cell-ECM interactions generating the supracellular fluidity underlying chicken feather follicle patterning (Palmquist et al. 2022). Second, integrin binding to some ligands can stimulate cell motility. For example, inhibiting integrins reduces the motility of outer epithelial cells driving branching morphogenesis of mouse embryonic salivary glands (Hsu et al. 2013). Third, integrins could reduce interfacial tension at cell-matrix contact surfaces, since integrins mediate cell spreading on many ECM substrates (Janmey et al. 2021). Though seemingly counterintuitive because integrin-mediated adhesions commonly enhance actin polymerization and myosin contraction to increase cortical tension, this contraction would be counterbalanced by mechanical resistance from integrin-bound ECM to increase effective adhesion energy and reduce net interfacial tension. Loss of β integrin disrupts *Drosophila* egg chamber morphogenesis by causing basal constriction and internalization of prefollicle cells (Lovegrove et al. 2019). This mechanism is also critical for the correct sorting pattern of primary human mammary gland epithelial cells and the budding morphogenesis of mouse embryonic salivary glands (Cerchiari et al. 2015, Wang et al. 2021a).

## OUTLOOK

There are many open questions in tissue morphogenesis. For example, despite increasing appreciation of the roles of ECMs in tissue morphogenesis, their dynamics, mechanical properties, and even composition often remain elusive. In most cases, we do not yet understand how biochemical and biomechanical signals are integrated to effect cell decisions. Nevertheless, there are many examples of their crosstalk. For example, the fibrillin matrix controls the bioavailability of TGFβ and BMP, whereas basement membrane perlecan can release endorepellin with antiangiogenic effects (Farach-Carson & Carson 2007, Ramirez & Sakai 2010). Mechanical cues from the ECM and cell adhesion can also influence cell metabolism, though this regulation is poorly understood (Romani et al. 2021).

This is an exciting time to study tissue morphogenesis given the advent of many enabling technologies. Live fluorescence microscopy will continue to be important because of the dynamic nature of morphogenesis. Multiview light-sheet microscopy has enabled in toto imaging of the mouse embryo from gastrulation to early organogenesis at single-cell resolution (McDole et al. 2018). Analysis of large-scale image data will undoubtedly benefit from deep learning–based solutions such as Cellpose for segmentation and methods for cell tracking (Malin-Mayor et al. 2023, Stringer et al. 2021). High-throughput sequencing approaches, including single-cell RNA-seq, ATAC-seq, CUT&RUN, and CUT&Tag, have facilitated unbiased cell type identification and gene regulation analysis (Kaya-Okur et al. 2019, Skene & Henikoff 2017, Tanay & Regev 2017). Spatial transcriptomics, for example, MERFISH and seqFISH, have enabled transcriptional expression analyses with preserved spatial contexts (Chen et al. 2015, Eng et al. 2019). Expanding CRISPR-based genome editing tools have enabled robust gene perturbation as well as knockin of mutations or functional tags (Wang & Doudna 2023). Targeted protein degradation, inducible gene activation or repression, and optogenetics provide valuable perturbation tools with increased spatiotemporal precision (Gao et al. 2016, Wittmann et al. 2020, Wu et al. 2020, Yavitt et al. 2023). Lastly, recent development of micro-oil droplets, micro hydrogel beads, and optical or magnetic tweezers have enabled biophysical characterizations in developing tissues (Bambardekar et al. 2015, Campàs et al. 2014, Doubrovinski et al. 2017, Vorselen et al. 2020).

Tissue morphogenesis research will demand next-generation tools and conceptual frameworks. We particularly need more scalable biophysical tools for systemic measurements of tissue rheological properties over time. While the DITH framework is useful for cellular tissues, it is difficult to incorporate ECMs. Some successful attempts to integrate cells and ECM in modeling are still limited to simple scenarios (Fiore et al. 2020). It will be of great interest to develop generalizable frameworks that incorporate both cells and ECMs with diverse mechanical properties. A common challenge confronting all of us is to extract biological insights from big data. We believe the key here is to combine big data and predictive modeling to generate hypotheses and to experimentally test them. In some cases, we could apply the understanding-by-building principle by reconstituting parts of the complex morphogenesis, as demonstrated recently (Wang et al. 2021a). Understanding the principles of tissue morphogenesis should ultimately enable rational design of tissues with desired shapes and functions.
